# The Attitude of Great Writers towards Disease

**Published:** 1892-08-06

**Authors:** 


### The Attitude of Great Writers Towards Disease.

### VIII.—DICKENS.

Of all our novelists Dickens has done the most to awaken sympathy for the desolate and despised. It is so much an axiom with the world at large in the present day that all, even the outcasts of society, have a claim on our civilisation for pity and succour, that it is not always remembered how little this claim was allowed forty or fifty years ago. It was not that no attempts had been made to invite attention to the wants of the people. A flood of literature was pouring forth, directed against the barriers which served to confine the blessings of peace and plenty to one small section of the nation. The pioneers of all the modern movements of social reform were already astir, and the most earnest minds of the century were among their number. But the public remained indifferent. Nothing, not actually brutal, was deemed too bad for the criminal classes, and thankfulness for their lot was unceasingly urged upon the poor, to the exclusion of any real attempt to understand their sufferings.

It was for this public that Charles Dickens began as a young man to paint the true life of the people. The names of some of those early sketches are sufficient to show their scope—"The Gin Shop," "Seven Dials," "The Criminal Courts," "The Pawnbroker's," "Visit to Newgate," "Greenwich Fair," &c. There is no attempt to dogmatise, to explain, or reform. The object is to show; and from first to last Dickens lent his genius to this task of showing. The rich were to be shown the poor, and taught that the same passions for good or evil stirred rich and poor alike. The happy and prosperous were to behold the sufferers and failures of this life, and learn that heroism, purity, and truth are flowers that even the dunghills of modern civilisation cannot altogether poison. One of these early sketches, relating a visit to a hospital, may be quoted as an example of his method. A brutal fellow, accused of fatally wounding his paramour, is taken to the wards of the hospital to be identified by her. It was evening. "The dim light which burnt in the spacious room increased rather than diminished the ghastly appearance of the hapless creatures in the beds, which were ranged in two long rows on either side. On every face was stamped the expression of anguish and suffering. The object of the visit was lying at the upper end of the room. Her face bore deep marks of the ill-usage she had received, her breathing was short and heavy, and it was plain to see that she was dying fast. The magistrate nodded to the officer to bring the man forward. After a brief pause the nature of the errand was explained and the oath tendered. 'Oh, no, gentlemen,' said the girl; 'no, gentlemen, for God's sake! I did it myself—it was nobody's fault—it was an accident. He didn't hurt me; he wouldn't for all the world. Jack, dear Jack, you know you wouldn't.' Her sight was fast failing her, and her hand groped over the bedclothes in search of his. . . . She grasped his arm tightly, and added in a broken whisper, 'I hope God Almighty will forgive me all the wrong I have done and the life I have led. God bless you, Jack. Some kind gentleman take my love to my poor old father,' and so died."

Here is, no doubt, the original of Nancy, if not of Bill Sykes. Here, too, is the early faith of Dickens in the triumph of love over evil in the power of suffering. There is nothing new in his philosophy. Many before him had tried to enforce the same lesson, but none had penetrated as he did into every house in the kingdom, uniting all classes alike in sympathy for the sorrows of the strolling player's child or the lonely terrors of the workhouse boy. Here lay the great gift of Dickens. He could bring the public over to his side. No reader of "Hard Times" could ever feel indifferent afterwards to the great suffering manufacturing population, judged in those days so harshly because so ignorantly; no reader of "Little Dorrit" or "Pickwick" could retain so comfortable a conviction that penal laws were necessary for debtors; no one could follow Betty Higden in that long wandering, which was mercifully ended by death, without doubting the wisdom of the nation in closing honourable lives of work by a dishonoured captivity.

But to the last the sufferings which laid most hold on Dickens, which through him laid most hold on his readers, were those of children. Who ever dreamt of taking childhood's troubles seriously before Dickens? Champions had been found indeed to plead that eighteen hours work a day for young children was excessive; and then, as now, physical torture when revealed awakened fitful bursts of indignation. But Dickens was the first to understand and lay bare the slow torture of neglect and contempt to which the young are so often exposed in workhouses, in schools, in loveless homes, or in no homes at all. Doubtless much of his rare sympathy with the troubles of childhood owed its origin to a bitter personal experience, but how few in the height of fame have turned to such noble account their early years of unhappiness. He made no secret of the fact that the early struggles of David Copperfield were painted from a vivid recollection of his own childhood. The following passages show how keen was his perception of a childish sorrow entirely distinct from those physical needs which many persons imagine must bound a child's horizon.

"No words can express the secret agony of my soul as I sunk into this companionship. The deep remembrance of the sense I had of being utterly without hope now; of the shame I felt in my position; of the misery it was to my young heart to believe that day by day what I had learned and thought, and delighted in, and raised my fancy and my emulation up by, would pass away from me little by little, never to be brought back any more, cannot be written. . . . From Monday morning to Saturday night I had no advice, no counsel, no encouragement, no consolation, no assistance, no support of any kind from anyone that I can call to mind, as I hope to go to Heaven." It is hardly too much to say that all the guilds, institutes, and working homes which aim now at befriending the lads of London owe their first impulse to the sympathy inspired by the lonely little bottle washer.

Every phase of childish suffering passes before us in his novels; the terrors of actual privation and brutality are exemplified in Smike and his fellow-sufferers in Dotheboy's Hall, and the pathetic figure of the little Marchioness, while Paul Dombey, killed by the very excess of his father's pride and anxiety for his future, showed that the author's sympathies could embrace also the sorrows of the rich and over-educated. Tiny Tim shows us the pains of poverty and disease sinking to nothing in the blessings of family love, and Oliver Twist is a beautiful revelation of innocence unsullied by contact with evil. Hardly one of his books could be named which has not its picture of childhood's troubles, small or great. But no child among them all took so powerful a hold on his readers as little Nell. Her death, long anticipated, was felt at length almost as a national calamity, and it is told of Daniel O'Connell that, having read the newly-issued number where her death was described, he sobbed aloud, "He should not have killed her! He should not have killed her! She was too good!" and threw the book out of the window.

It may be objected that Dickens sometimes touches a note almost too secret and sacred for purposes of art. Thackeray had this thought in his mind, when he says: "As for Tiny Tim, there is a certain passage in the book regarding that young gentleman, about which a man should hardly venture to speak in print or in public, any more than he would of any other affections of his private heart." But Dickens was not ashamed of his own pathos. Speaking in the person of Pip, he has said, "Heaven knows we need never be ashamed of our tears, for they are rain upon the blinding dust of earth overlying our hard hearts." Lord Cockburn was the mouthpiece of thousands when he wrote, after crying over Paul Dombey's death, "I felt my heart purified by those tears, and blessed and loved you for making me shed them."

For it was in no spirit of vain sentimentality that Dickens touched the spring of tears in his readers. The generation which wept over his pages for the sufferings of the poor and despised, was the generation which first grappled seriously with the glaring evils of our civilisation, and the share of Charles Dickens in the great wave of enthusiasm which still stirs men's hearts can hardly be over-rated, yet is often forgotten.

